# Large Animal Models of Vascularized Composite Allotransplantation: A Review of Immune Strategies to Improve Allograft Outcomes

**DOI:** 10.3389/fimmu.2021.664577

**Published:** 2021-06-30

**Authors:** Abraham J. Matar, Rebecca L. Crepeau, Gerhard S. Mundinger, Curtis L. Cetrulo, Radbeh Torabi

**Affiliations:** ^1^ Department of Surgery, Emory University School of Medicine, Atlanta, GA, United States; ^2^ Department of Surgery, Division of Plastic and Reconstructive Surgery, School of Medicine, Louisiana State University Health Sciences Center, New Orleans, LA, United States; ^3^ Department of Surgery, Division of Plastic Surgery, Massachusetts General Hospital, Boston, MA, United States; ^4^ Center for Transplantation Sciences, Massachusetts General Hospital, Boston, MA, United States; ^5^ Shriner’s Hospital for Children, Department of Plastic and Reconstructive Surgery, Boston, MA, United States

**Keywords:** vascularized composite allograft, large animal model, transplantation tolerance induction, cellular therapies, *ex vivo* perfusion

## Abstract

Over the past twenty years, significant technical strides have been made in the area of vascularized composite tissue allotransplantation (VCA). As in solid organ transplantation, the allogeneic immune response remains a significant barrier to long-term VCA survival and function. Strategies to overcome acute and chronic rejection, minimize immunosuppression and prolong VCA survival have important clinical implications. Historically, large animals have provided a valuable model for testing the clinical translatability of immune modulating approaches in transplantation, including tolerance induction, co-stimulation blockade, cellular therapies, and *ex vivo* perfusion. Recently, significant advancements have been made in these arenas utilizing large animal VCA models. In this comprehensive review, we highlight recent immune strategies undertaken to improve VCA outcomes with a focus on relevant preclinical large animal models.

## Introduction

Over the past twenty years, notable surgical developments have advanced the field of vascularized composite tissue allotransplantation (VCA), making it a viable treatment for patients with major defects involving multiple layers of tissue including hand, face, penis, and laryngeal transplantation, among others (1–5). As in solid organ transplantation, VCA must overcome a significant immune barrier to provide meaningful, long-term benefit. Strategies to regulate the immune system in favor of VCA tolerance have important clinical implications as there is a significant risk of acute and chronic rejection after VCA (6). Further, unlike solid organ grafts, VCAs primarily serve to enhance quality of life rather than provide life-sustaining organ function. Consequently, there is risk to exposing patients to the untoward effects of lifelong immunosuppressive regimens required to prevent VCA rejection, including medication side effects, infectious complications and risk of malignancy.

Given that VCA is a relatively new clinical endeavor compared to solid organ transplantation, there are a variety of proven strategies to minimize immune mediated damage of solid organ allografts that can be translated to VCA. Historically, large animals have proven to be an important model in which to test the clinical translatability of immune modulating approaches in transplantation given the limited success in translating findings from murine model systems directly to humans. In particular, large animals are a highly relevant model for VCA studies owing to their anatomic and physiologic similarities to humans (7). Early large animal VCA studies overcame technical barriers to establish clinically relevant models (8–12). Currently, a major area of focus is optimizing approaches to control the alloimmune response with the aim of clinical translation. In this review, we highlight recent advancements in immune modulating strategies to improve VCA outcomes in relevant preclinical large animal studies.

## Methods

A PubMed search was conducted using the following search terms: “vascularized composite allotransplantation”, “composite tissue allotransplantation”, “large animal models”, “nonhuman primates”, “cynomolgus”, “rhesus macaque”, “miniature swine”, “canine”, “tolerance”, “hematopoietic stem cells”, “mixed chimerism”, “co-stimulation blockade”, “mesenchymal stem cells”, “dendritic cells”, “chronic rejection”, “local immunosuppression” and “*ex vivo* perfusion”. Inclusion criteria consisted of articles describing studies involving non-human primates (NHP), swine, or canine models of VCA that investigated an immune modulating approach in VCA. Articles that focused on the technical aspects of model development related to VCA were excluded and were beyond the scope of this review. In total, 20 articles describing immune strategies to improve VCA outcomes in large animals were reviewed.

## Hematopoietic Cell Transplantation

Hematopoietic cell transplantation (HCT), either in the form of infused bone marrow (BM), cytokine “mobilized” cells, or vascularized bone marrow (VBM), can induce a state of mixed chimerism, in which donor and host derived cells coexist in a state of tolerance without a host *versus* graft allo-response (rejection) or graft *versus* host disease (GVHD). Following the induction of mixed chimerism and immune tolerance, transplantation of a subsequent donor allograft is accepted without additional immunosuppression (IS) (13–15). Stable multi-lineage mixed chimerism has been readily achieved in murine transplant model systems, however clinical attempts at translating these protocols has only consistently achieved transient donor chimerism (14). Despite not achieving long-lasting macro-chimerism, transient mixed chimerism is adequate to facilitate tolerance to a subsequent donor allograft if transplanted during the interval of transient chimerism (14).

As in humans, the majority of HCT regimens in NHP induce transient mixed chimerism, making NHP an excellent pre-clinical model for mixed chimerism studies (16, 17). While early VCA studies in primates strove to generate technically attainable and immunologically relevant models (8, 11, 18), recent studies have sought to utilize donor BM, both vascularized and infused marrow, to modulate the alloimmune response and prolong graft survival (19–21) (**Table 1**). Barth et al. (19) assessed the immunological impact VBM in a VCA model (facial segment), in which cynomolgus monkeys received VCA grafts with or without VBM, which incorporated the donor mandible. All recipients received IS in the form of tacrolimus (goal whole blood level ≤ 30 ng/mL) and mycophenolate mofetil (MMF). A previous study demonstrated that higher levels of tacrolimus (30 ng/mL – 50 ng/mL) were associated with high rates of post-transplant lymphoproliferative disease (PTLD) in recipients of VBM (25). Recipients receiving VCA grafts alone (without VBM) universally underwent acute rejection by post-operative day (POD) 15 and graft loss by POD 42. In contrast, the addition of VBM delayed the onset of acute rejection and significantly prolonged graft survival to 267 – 462 days. Interestingly, intermittent low-level (1-12%) donor macro chimerism could be detected in the peripheral blood of VCA + VBM recipients, but not VCA alone recipients. The addition of VBM, however, did not induce true VCA tolerance, as grafts were promptly rejected once IS was discontinued (19). However, this study served as proof of principle that the intragraft VBM component conferred an immunological benefit on VCA survival, since confirmed in murine models (26, 27).

**Table 1:** Bone marrow and hematopoietic cell transplantation.

Immune Strategy	Large Animal Model	VCA model	IS/Regimen	Experimental Groups	Results/Conclusion	Reference
Vascularized Bone Marrow	Cynomolgus Macaques	Facial segment VCA	Tacrolimus/MMF	Group 1 – VCA alone without VBM + MMF/Tacrolimus	Group 1 – survival < 42 days	(19)
				Group 2 – VCA + VBM (in the form of donor mandible) + MMF/Tacrolimus	Group 2 – survival 267 – 462 days. VBM induces transient chimerism and inhibits VCA rejection until IS withdrawal.	
HCT/mixed chimerism	Miniature Swine	Fasciocutaneous VCA	CD3 Immunotoxin, 1 Gy TBI, 30-day course of CyA	Group 1 – Untreated controls	Group 1 – survival 6 days	(22)
				Group 2 – Delayed VCA after HCT and chimerism induction	Group 2 – 115 – 504 days	
				Group 3 – Simultaneous VCA and HCT	Group 3 – 79 – 486 days. Long term tolerance to VCAs can be achieved through mixed chimerism induction	
Infused Bone Marrow	Cynomolgus Macaques	Facial segment VCA	Tacrolimus/MMF	Group 1 – VCA alone without BMC	Groups 1 and 2 – Infused BM confers no advantage on VCA survival	(20)
				Group 2 – VCA + BMC		
HCT/mixed chimerism	Canine	Rectus VCA	1 or 2 Gy TBI	Group 1 – Simultaneous bone marrow + VCA + MMF + CyA	Group 1 – 100% VCA survival 62-69 weeks after cessation of IS	(10)
			MMF, CyA	Group 2 – VCA + MMF + CyA	Group 2 – 3 out of 4 animals rejected VCA after cessation of IS. Simultaneous establishment of mixed chimerism and VCA tolerance is feasible.	
			Minor-mismatched BMT			
HCT/mixed chimerism	Canine	Rectus VCA	4.5 Gy TBI, MMF, CyA	Group 1 – BMT + VCA + MMF + CyA	Group 1 – 3 out of 4 animals rejected BM and VCA at 5-7 weeks post-transplant	(23)
			HCT (cytokine mobilized *vs.* BMT)	Group 2 – cytokine mobilized HCT +VCA + MMF + CyA	Group 2 – 100% acceptance of VCA and long term tolerance. Cytokine mobilized stem cells are superior to bone marrow for mixed chimerism induction and VCA tolerance but associated with increased incidence of GvHD.	
HCT/mixed chimerism	Miniature Swine	Vascularized skin flap	CD3 Immunotoxin, 1 Gy TBI, 30-day course of CyA	Group 1 – MHC class I mismatched VCA transplant	Group 1 – Rejected VCA skin	(24)
				Group 2 – MHC class II mismatched VCA transplant	Group 2 – Were tolerant of all VCA components. MHC class I matching is necessary for skin tolerance and long-term VCA tolerance.	
Delayed BMT	Cynomolgus Macaques	Orthotopic upper extremity and heterotopic partial face VCA	Anti-thymocyte globulin (ATG), tacrolimus, MMF and methylprednisolone, BMT	6 animals underwent VCA transplant followed by induction regimen and delayed BMT 2 months later	All animals developed acute rejection within 2-4 weeks after VCA transplant. Following BMT, mixed chimerism failed to develop and VCAs underwent irreversible graft loss. Delayed tolerance induction protocol not capable of inducing tolerance to VCA.	(21)

IS, immunosuppression; RBC, red blood cells; VCA, vascularized composite allotransplant; BMC, bone marrow cells; BMT, bone marrow transplant; CyA, cyclosporin A; GvHD, graft vs. host disease; HCT, hematopoietic cell transplant; MHC, major histocompatibility complex; MMF, mycophenolate mofetil, TBI, total body irradiation, VBM, vascularized bone marrow.

Two subsequent studies evaluated the use of infused bone marrow as the source of donor hematopoietic stem cells (HSC) (20, 21). Unlike VBM, Brazio et al. (20) reported no survival benefit when infused BM was used in conjunction with tacrolimus and MMF. This despite a significantly higher cell yield (~6x) when compared to VBM and presumably more HSCs (20). A caveat to these studies is the lack of preconditioning used, such as T cell depletion agents or irradiation, which have been shown to augment HSC engraftment and facilitate mixed chimerism. Lellouch et al. (21) utilized a delayed tolerance induction protocol which has previously been used successfully to induce transient mixed chimerism and confer tolerance to renal and lung allografts in NHP (15, 28). NHP underwent upfront VCA transplantation utilizing a conditioning regimen of anti-thymocyte globulin (ATG), tacrolimus, MMF and methylprednisolone. This was followed by a two-month period of IS, after which NHP underwent BM transplantation with donor BM cells cryopreserved at the time of operation. This delayed induction protocol supposes that recipient conditioning and BM transplantation at a time point removed from the peri-transplant period and associated inflammatory milieu is advantageous for the generation of chimerism and tolerance. Unlike previous studies in kidney and lung transplant utilizing this model, transient mixed chimerism was not achieved in this study, and VCA grafts were quickly rejected following withdrawal of IS. The authors note that unlike kidney and lung allografts, VCA allografts may have been compromised by acute rejection episodes in the time interval between VCA and BM transplant despite ATG depletion and triple IS. Activation of the allo-immune response during this interval may have impeded chimerism induction post BMT (21).

Altogether, these studies underscore the substantial immunogenicity of VCA allografts in NHP compared to solid organ grafts, likely as a result of skin antigens. Successful translation of mixed chimerism and tolerance protocols in NHP VCA models will require additional strategies aimed at immune modulation to prevent both acute and chronic rejection. While acute rejection is more commonly observed after clinical VCA, chronic rejection remains a poorly understood and poorly defined entity due to the paucity of transplants performed worldwide (29). Several large animal models have proven to demonstrate features of chronic rejection consistent with clinical VCA (12, 30). In a cynomolgus macaque model of free fibula VCA, 2/5 animals developed evidence of chronic rejection including vessel occlusion by intimal proliferation, transplant vasculopathy and graft fibrosis (12). Interestingly, the development of chronic rejection did not appear to be associated with prior episodes of acute rejection, suggesting a possible distinct pathway for the development of chronic rejection in VCA. In a subsequent NHP model of heterotopic face transplantation, five animals were weaned off IS after 200-day survival to assess the histological features of chronic rejection (30). All five animals progressed to graft loss, characterized by tertiary lymphoid follicles, neointimal proliferation, and vessel wall fibrosis. While there is evidence linking the production of alloantibody to chronic rejection in solid organ transplantation, neither NHP VCA model revealed any connection between alloantibody formation and chronic VCA rejection (31). Further, both NHP models demonstrated that chronic graft rejection can be present without overlying skin changes, supporting the routine use of deeper biopsies to monitor for evidence of chronic rejection. Collectively, these observations of chronic rejection in NHP VCA models have since been validated in clinical reports of chronic VCA rejection, further supporting the use of large animal VCA models, particularly NHP (32–34).

Unlike NHP, swine and canine models are generally conducive to the induction of stable mixed chimerism and therefore long-term tolerance to VCA grafts (22–24, 35). Based on these consistently achievable results, swine and canine VCA models allow for mechanistic insights into VCA tolerance including the effect of MHC antigen matching on skin tolerance, the temporal relationship between BMT and VCA necessary for tolerance induction, and the effectiveness of BM *versus* cytokine mobilized HSC in VCA tolerance induction. Shanmugarajah et al. (24) evaluated the effect of MHC class I *versus* class II mismatching on skin tolerance induction in a swine HCT + VCA model and found that MHC class I matching was necessary for skin tolerance and long-term VCA tolerance despite the induction of stable mixed chimerism (24). This result has important clinical implications, as currently, clinical VCAs do not undergo MHC antigen matching because of the small pool of potential donors. Moving forward, careful attention to matching VCA donor and recipients for MHC class I antigens may potentially improve long term VCA outcomes.

In a canine model of HCT + VCA, Mathes et al. (10) described acceptance of VCA grafts between 52-90 weeks following establishment of mixed chimerism. Given that the majority of VCA grafts used clinically are deceased donor grafts, such a prolonged period between HCT and VCA transplant is not usually feasible. Therefore, Mathes et al. (35) tested the hypothesis that established immunological tolerance was not required for VCA acceptance, and that simultaneous establishment of mixed chimerism and VCA tolerance was possible. Four dog leukocyte antigen (DLA) identical (minor antigen mismatched) transplants were performed, and recipients underwent 200 cGy of radiation. Three of four recipients developed stable mixed chimerism and all four animals remained tolerant to all components of the VCA despite withdrawal of IS. This result served as proof of principle that simultaneous establishment of mixed chimerism and VCA tolerance was possible, increasing the potential clinical translatability of HCT + VCA protocols (35). Finally, Chang et al. (23) evaluated whether this protocol could be extended across an MHC haploidentical barrier and whether either BM or cytokine mobilized peripheral stem cells (CMPSC), *via* granulocyte-colony stimulating factor (G-CSF), conferred any advantage. Eight total haploidentical canine pairs underwent simultaneous HCT and VCA transplantation – four received BM and four received CMPSC. While only one animal (25%) in the BM group became chimeric and accepted its VCA graft long term, all four animals in the CMPSC group accepted their stem cell grafts and maintained immune tolerance to VCA long term (one animal eventually rejected its stem cell graft but remained tolerant to the VCA). Interestingly, there was a high incidence of GVHD in the CMPSC group (75%), which is not completely unexpected given the haploidentical MHC barrier and the higher dose of irradiation (450 cGy) used to condition the recipients (23). These results are consistent with a previous study across haploidentical MHC barriers in miniature swine utilizing cytokine mobilized peripheral stem cells (22).

The induction of tolerance to VCA allografts is the ideal scenario as it would minimize the risks conferred by long-term IS. A preliminary study in five human recipients of VCA suggested that infusion of BM cells was well tolerated and facilitated maintenance IS with tacrolimus monotherapy, a significant improvement compared to standard triple IS (36). While swine and canine HCT + VCA models allow for mechanistic insights, NHP models more consistently reflect the clinical scenario in which transient, and not stable, chimerism is achievable. Although it is unclear why the immune threshold to achieve stable mixed chimerism in NHP is higher than for swine or canines, a contributing factor may be the increased frequency of memory T cells in primates (37). Further refinement of these HCT protocols is necessary prior to clinical translation, which may include several of the strategies discussed below.

## Costimulation Blockade

Costimulation blockade (CoB) has recently come to the forefront of solid organ transplantation as a viable alternative to standard calcineurin inhibitor (CNI) therapy. Belatacept, a CTLA4-Ig fusion protein was recently shown to confer improved graft and patient survival compared to CNI in renal transplant patients (38). Despite these advantages, increased rates of early acute cellular rejection have impeded the use of CoB based regimens. A major contribution to CoB refractory rejection appears to be the relative resistance of memory CD8+ T cells to CoB given the lack of requirement of costimulation signaling for activation. Nevertheless, CoB based therapy blocking either the CD28-CD80/86 or CD40-CD154 pathways has shown promising results in murine VCA models (26, 27, 39). There have been few studies of CoB based regimen in large animals (40–42) (**Table 2**). Wachtman et al. (40) first evaluated the efficacy of combination CTLA4-Ig + tacrolimus compared to tacrolimus alone in a Yucatan miniature swine model and found that the addition of CTLA4-Ig significantly increased survival of grafts from a mean of 31 days to > 150 days. Atia et al. (41) hypothesized that the addition of Th17 blockade to a CoB based regimen would limit T cell infiltration and may prolong graft survival. Treatment with ustekinumab (anti-IL12/23) and secukinumab (anti-IL17A) was shown to decrease skin T cell infiltration and expression of IL17A but was not associated with prolonged graft survival. In the most expansive study to date of CoB based regimens in large animal VCA model, Freitas et al. (42) evaluated the use of several CoB agents, including CTLA4-Ig and Belatacept, in a NHP study comparing CNI to CoB based regimens. Overall, the use of CoB was associated with improved rejection free survival compared to tacrolimus, justifying further preclinical studies of CoB based therapy for VCA. One hypothesis for this outcome was the prevention of donor specific antibody (DSA) formation in the CoB group, a known therapeutic effect of CoB (43). This study also highlighted the careful balance required between control of rejection and protective immunity as several animals required euthanasia with high cytomegalovirus (CMV) viral loads due to failure to thrive. CMV prophylaxis for high-risk individuals is standard practice in solid organ transplantation and should be an important consideration in the clinical VCA setting. Another important observation in this study was the association between sirolimus and lack of VCA engraftment. The authors initially used sirolimus in combination with CoB based on mechanistic studies that indicated a synergistic effect between the two (44). However, sirolimus was associated with healing complications resulting in lack of VCA engraftment. This observation in a clinically relevant NHP model suggests that sirolimus may not be an appropriate agent to use in the immediate post-operative VCA setting. Initial treatment with tacrolimus with conversion to sirolimus ameliorated the wound healing complications. This study represents the first experience with a CoB based regimen in a NHP VCA model (42). Further studies are necessary to optimize a CoB based regimen for VCA.

**Table 2:** Costimulation blockade.

Large Animal Model	VCA model	IS	Experimental Groups	Results/Conclusions	Reference
Yucatan miniature swine	Hind limb VCA	Leukocyte Depletion: TBI and TI	Group 1 - no treatment; Group 2 - Tacrolimus only; Group 3 - irradiation, BMT, and FK506; Group 4 - received Tacrolimus and CTLA4-Ig	Group 1 survival: 5-8 days	(40)
		Cell infusion: Animal groups received 15, 30, or 60 million cells per kilogram of whole unmodified BM.		Group 2 survival: 30 – 32 days (only skin and muscle rejected)	
		Immunosuppression: CTLA4Ig.		Group 3 survival 5-53 days (only skin rejected).	
				Group 4 survival: > 150 days (skin survival in 2/3 animals > 150 days).	
				Addition of CTLA4-Ig to calcineurin inhibitors significantly prolonged survival of VCA grafts.	
Rhesus Macaque	Radial forearm VCA	Belatacept, Steroids, Ustekinumab (Anti-IL12/23, Secukinumab (anti-IL17A)	Group 1 - Belatacept and steroids; Group 2 - Belatacept, Ustekinumab with steroid taper; Group 3 - Belatacept, Secukinumab with steroid taper.	Group 1 survival: 10 days	(41)
				Group 2 survival: 10.33 days	
				Group 3 survival: 11 days	
				Ustekinumab and secukinumab were associated with decreased T-cell infiltration and IL-17a expression in the allograft but had no impact on VCA survival in the setting of Belatacept and steroids.	
Rhesus Macaque	Radial forearm VCA	Belatacept/CTLA4Ig *vs.* Tacrolimus	Group 1 – Tacrolimus; Group 2 – CTLA‐4Ig, alefacept, sirolimus; Group 3 – CTLA‐4lg, alefacept, tacrolimus conversion to sirolimus; Group 4 – Belatacept, alefacept, tacrolimus conversion to sirolimus; Group 5 – Belatacept, tacrolimus conversion to sirolimus.	CoB was superior to calcineurin inhibitors in prolonging VCA survival.	(42)
				Sirolimus was associated with healing complications resulting in lack of VCA engraftment.	

IS, immunosuppression; TBI, total body irradiation; TI, thymic irradiation; RBC, red blood cells; VCA, vascularized composite allotransplant; BM, bone marrow; CoB, co-stimulation blockade; Ig, immunoglobulin.

## Cellular Therapies

Cellular therapies have long been an area of study as an adjunct to traditional IS in solid organ transplantation. Various regulatory cell populations have been studied in large animal models of transplantation including regulatory T cells, bone marrow derived mesenchymal stem cells (BM-MSCs), and dendritic regulatory cells (DCregs) (45–47). Tolerance induction *via* traditional bone marrow transplantation can be complicated by significant risks including GVHD as well as the morbidity of the conditioning regimen. In comparison, cellular therapies can modulate the alloimmune response in a more directed manner, minimizing collateral effects. This is especially relevant in the setting of VCA in which patient morbidity is of utmost concern based on the nature of VCA as a life-enhancing graft.

Kuo and colleagues (48–50) evaluated the efficacy of bone marrow derived MSCs in swine VCA models (**Table 3**). First, donor derived BM-MSCs were administered alone or incorporated into a traditional BMT regimen to improve VCA survival in a heterotopic hind-limb model (50). When compared to untreated controls, multiple BM-MSCs infusions alone without IS had a modest, but significant effect on survival (9-14 days *vs.* 15-25 days). However, when BM-MSCs were used as part of a BMT protocol that included irradiation, cyclosporin A (CyA), and BMT, the effect was amplified. Animals receiving the BMT regimen alone experienced VCA survival of 13-57 days. The addition of BM-MSCs extended survival to >200 days. Animals tolerant of their VCA allografts demonstrated increased regulatory T cells in the peripheral blood, a known byproduct of BM-MSC infusion (49, 50). Analysis of donor VCA skin revealed the presence of donor BM-MSCs, indicating that infused MSCs homed to the graft and may have provided an additional local protective effect. Similarly, in a swine hemi-facial allotransplant model, BM-MSCs had only a modest, but not statistically significant, effect on allograft survival (mean survival time [MST] – 34 days) compared to untreated controls (MST – 9 days) (48). However, the addition of transient IS with CyA augmented the effect of MSCs, significantly prolonging graft survival. Animals treated with MSCs again demonstrated increased Tregs in the peripheral blood and graft, as well as decreased expression of proinflammatory cytokines (TNF-a) in circulation. These results in a large animal VCA model support murine findings that BM-MSCs are a novel cellular therapy capable of suppressing the alloimmune response and prolonging VCA survival in the setting of concomitant IS.

**Table 3:** Cellular therapies.

Large Animal Model	VCA model	IS	Experimental Groups	Results	Conclusion	Reference
Swine	Heterotopic hind-limb VCA	Irradiation, BMT, Cyclosporine, MSCs	Group 1 – untreated controls.	Group 1 Survival – 9-14 days	Donor MSCs augment the effect of BMT and significantly prolong VCA survival.	(50)
			Group 2 - received MSCs alone (given on days -1, +3, +7, +14, +21).	Group 2 survival – 15-25 days		
			Group 3 - received cyclosporine A.	Group 3 survival – 28-45 days		
			Group 4 - received preconditioning irradiation (day -1), BMT (day +1), and CyA.	Group 4 survival - 13-57 days		
			Group 5 - received irradiation (day -1), BMT (day +1), CyA, and MSCs (days +1, +7,+14).	Group 5 survival – > 200 days		
Swine	Heterotopic hind-limb VCA	Irradiation, Cyclosporine, MSCs	Group 1 – untreated controls.	Group 1 Survival – 9-14 days	Donor MSCs prolong VCA survival in the setting of irradiation/CyA	(49)
			Group 2 – received MSCs alone (on days -1, +3, +7, +14, and +21).	Group 2 survival – 15-25 days		
			Group 3 – received cyclosporine A.	Group 3 survival – 28-45 days		
			Group 4 received irradiation (on day -1), MSCs (days +1, +7, +14, and +21), and cyclosporine A.	Group 4 survival – > 120 days		
Swine	Orthotopic hemi-facial VCA	Cyclosporine, MSCs	Group 1 – untreated controls.	Group 1 Survival – 7-28 days	Donor MSCs + transient CyA significantly prolong VCA survival.	(48)
			Group 2 – received MSCs alone (on days -1, +1, +3, +7, +14, and +21).	Group 2 survival – 17-38 days		
			Group 3 – received cyclosporine A.	Group 3 survival – 36-48 days		
			Group 4 received MSCs (days -1 +1, +3, +7, +14, and +21), and cyclosporine A.	Group 4 survival – 42-87 days		

IS, immunosuppression; MSC, mesenchymal stem cells; CyA, cyclosporine; BMT, bone marrow transplantation; VCA, vascularized composite allotransplant.

The use of MSCs in clinical VCA transplant may be an exciting strategy to improve outcomes. The safety of MSCs has already been demonstrated in various clinical trials in solid organ transplantation, which could pave the way for their use in VCA (51, 52). However, there remain outstanding questions that need to be further explored prior to applying MSC-based therapies to human VCA patients including their mechanisms of action in preventing rejection *in vivo* as well as the fate of MSCs following infusion. Interestingly, murine studies suggest that MSCs are quickly trapped within the pulmonary circulation after intravenous administration and only a small proportion of MSCs reach the systemic circulation and peripheral tissues (53, 54). For these reasons, the long-lasting immunomodulatory effect of MSCs is likely mediated through their ability to harness the effects of other cell types including Tregs. MSCs are capable of inducing Tregs through various mechanisms including TGF-B and indoleamine 2,3-dioxygenase (IDO) (55, 56). Confirming these murine findings in large animal models will be an important step in paving the way to clinical trials of MSCs in VCA.

DCregs are a population of bone marrow derived dendritic cells with immune suppressive properties (57). In murine models, DCregs are capable of inducing transplant tolerance through both peripheral and central mechanisms (57). Based on these tolerogenic properties, DCregs have been studied in early phase clinical trials of autoimmune disease and were shown to be safe (58, 59). Current trials of autologous and donor derived DCregs in kidney and liver transplantation are currently underway. DCregs have been generated in NHP using granulocyte-macrophage colony stimulating factor (GM-CSF) and GCSF mobilization followed by leukapheresis to capture CD14+ monocytes (46, 60). CD14+ monocytes were then cultured with GM-CSF, IL-4, vitamin D3, and IL-10 to generate DCregs. In a NHP model of kidney transplantation, both donor derived and autologous DCregs significantly prolonged renal allograft survival, providing a foundation for DCreg use in large animal VCA models (46, 60). Currently, preliminary studies are ongoing to produce swine DCregs for evaluation in a miniature swine VCA model (61). Elgendy et al. (61) generated swine DCregs from both bone marrow and peripheral blood sources. Using a similar culture strategy as above, swine DCregs were generated and found to have similar properties as NHP DCregs including poor allogeneic stimulation, reduced MHC class II expression, and low levels of costimulatory molecules (CD80/86) (61). *In vivo* studies utilizing these swine DCregs in a VCA model are forthcoming.

## Local Immunosuppression

The systemic effects of chronic IS can cause significant morbidity in VCA recipients and impair quality of life. Unlike solid organ allografts, VCAs are candidates for local interventions and delivery of IS given their easy accessibility. Local IS strategies, such as topical application of IS or the use of a local drug delivery system, could minimize systemic exposure of IS while maintaining sufficient levels at the graft site to prevent rejection. The mechanisms of local delivery of IS are not completely intuitive as much of the IS administered locally likely does not directly reach secondary lymphoid organs (62). Site specific delivery of IS may have alternative effects including inhibiting APC activation, mitigating transendothelial migration of activated leukocytes and prevention of ischemia reperfusion injury (62).

Clinically, there is anecdotal evidence that topical IS may be useful in the treatment of low-grade acute rejection, although there have been no randomized trials evaluating the efficacy of topical IS in VCA (63). Rodent models have suggested that local IS may be equally as efficacious as systemic IS in preventing VCA rejection (64, 65). In a rat hindlimb model, Gajanayake et al. (64) demonstrated the utility of an enzyme responsive tacrolimus-laden hydrogel to provide local IS at the graft site. The hydrogel is designed to release the drug (tacrolimus) upon encountering proteolytic enzymes, such as matrix metalloproteinases, produced during inflammation. A single local injection of this tacrolimus-laden hydrogel prolonged VCA survival > 100 days, compared to local tacrolimus alone (no hydrogel) (33.5 days) (64). In a different study by the same group, subcutaneous intragraft applications of a tacrolimus hydrogel prolonged graft survival as long as daily systemic tacrolimus, despite a four times smaller overall tacrolimus dose in the subcutaneous group (65).

Several large animal models have studied the efficacy of these local IS strategies (66–68) (**Table 4**). Mastroianni et al. (66) evaluated the use of slow-release topical formulations of CyA and tacrolimus in baboons by applying these formulations topically after allogeneic and xenogeneic split thickness skin grafts (STSG) as well as to the wound bed prior to skin grafting. In both scenarios, there was no improvement in STSG survival with the use of either topical CyA or tacrolimus, although histology revealed decreased inflammatory cell infiltrate in tacrolimus treated STSG. It is unclear whether the dosing used in this study was sufficient to significantly prolong graft survival (66).

**Table 4:** Local Immunosuppression.

Large Animal Model	VCA model	IS	Experimental Groups	Results/Conclusion	Reference
Yorkshire Swine	Gracilis musculocutaneous flap	Hydrogen Sulfide	Group 1 (control) – 3-hour ischemic period without perfusion.	H2S perfusion associated with decreased levels of injury biomarkers and proinflammatory cytokines including creatine kinase, LDH, and AST	(69)
			Group 2 – 3-hour ischemic period with an interim perfusion of H2S.		
Baboons	Skin	Slow-release (TyroSphere-encapsulated) topical formulations (cyclosporine or Tacrolimus)	Group 1 – Topical formulations applied directly to the STSGs only.	No graft survival benefit of topical treatment to either the STSG or wound bed	(66)
			Group 2 – Topical formulations applied to both the wound bed and the STSG.		
Swine	Gracilis musculocutaneous flap	Hydrogen Sulfide	Group 1 (control) – 3 hours of cold ischemic time without treatment.	Local pretreatment with H2S delayed the onset of rejection both by clinical and histopathological assessment.	(67)
			Group 2 –Allografts pretreated with hydrogen sulfide prior to 3 hours cold ischemic time.		
Swine	Forelimb VCA	Enzyme responsive, tacrolimus-eluting (TAC) hydrogel	Group 1 (control) – No treatment.	Group 1 – survival 6-7 days	(67)
			Group 2 – Low dose TAC hydrogel (49 mg).	Group 2 survival – 56-93 days	
			Group 3 – High dose TAC hydrogel (91 mg).	Group 3 survival – 24-42 days	
				Graft implanted TAC hydrogel allows for long term survival of orthotopic forelimb VCA in absence of systemic IS. Low dose treatment better tolerated than high dose treatment.	

IS, immunosuppression; H2S, hydrogen sulfide; STSG, split thickness skin graft; VCA, vascularized composite allotransplant; AST, aspartate aminotransferase; LDH, lactate dehydrogenase.

The role of ischemia reperfusion injury (IRI) in VCA outcomes is not yet completely understood, although several studies have suggested that IRI may contribute to acute rejection episodes, development of chronic rejection, and impede tolerance induction. Hydrogen sulfide (H2S) has previously been shown to avert the development of IRI in autologous swine VCA model (gracilis musculocutaneous flap) and was associated with significant decreases in skeletal muscle IRI biomarkers including creatine kinase, lactate dehydrogenase, and aspartate aminotransferase (AST) (69). The same group then attempted to translate these findings to an allotransplant model, in which allografts were pretreated with H2S prior to three hours of cold ischemia time (67). Recipients did not receive any systemic IS. Compared to untreated controls undergoing the same period of cold ischemia, H2S delayed the onset of rejection both by clinical and histopathological assessment, but the effect was most dramatic in the early postoperative period (day 6-8). Future studies may combine H2S graft pretreatment with traditional IS to determine if the effect of H2S can be augmented.

Finally, Fries et al. (68) utilized a similar enzyme responsive, tacrolimus-eluting (TAC) hydrogel as described above, in a swine limb VCA model. Orthotopic forelimb transplants were performed across one haplotype MHC barrier in miniature swine. Untreated controls rejected by POD 6. In comparison, two experimental groups received an injection of either low (49 mg per limb) or high (91 mg per limb) dose of TAC hydrogel on the day of transplant. Both treatment groups experienced prolonged graft survival times (low dose 56-93 days; high dose 24-42 days), although the group receiving low dose treatment fared clinically better than the high dose treatment group in which animals necessitated euthanasia due to failure to thrive. Whole blood levels of tacrolimus were not different between the two experimental groups, so the reason for the clinical differences is unclear. Nevertheless, such a significant prolongation of VCA survival in the absence of systemic IS is a notable achievement and serves as proof of principle that drug eluting hydrogel is a viable form of IS delivery in VCA. The authors noted that future studies will need to focus on optimizing measurement of graft delivered IS as well as restricting drug release to episodes of acute rejection while minimizing release in response to non-specific inflammatory insults (68).

Whereas local IS seems to efficiently control the ongoing process of T cell mediated rejection of the allogenic skin, there is little evidence that it has any impact on the humoral arm of the immune response. The advantage of local IS, which reduces the need for systemic IS, could be counterbalanced by its inability to inhibit the occurrence of chronic vascular rejection (chronic allograft vasculopathy), the main cause of VCA graft loss in the long term. For this reason, it is unlikely that local IS will serve as a replacement for systemic IS in VCA; rather it will most likely be utilized as an adjunct to reduce the amount of systemic IS required to temper the immunogenicity of VCA allografts.

## *Ex Vivo* Perfusion

The current standard of care for organ preservation is static cold storage at 4 degrees Celsius in University of Wisconsin (UW) solution*. Ex vivo* perfusion of organs has recently emerged as a tool to preserve the viability of time-sensitive organs, revitalize marginal organs, and immunomodulate grafts prior to transplantation (70, 71). Early-stage clinical trials are currently underway in solid organ transplantation including kidney (NCT03837197), liver (NCT03930459), heart (NCT00855712) and lung (NCT01365429). Due to the limited availability of donors and time sensitivity associated with VCA grafts, prolonging the period between procurement and revascularization while preserving viable tissue could significantly increase the donor pool and increase the indication for VCA. Further, as a result of the muscle component, VCA grafts are particularly susceptible to IRI. The ability to “optimize” VCA grafts on an *ex vivo* circuit could potentially diminish the degree of IRI after revascularization. Large animal preclinical studies are especially valuable in the area of *ex vivo* perfusion for logistical reasons, mainly the size similarities between large animal and human organs.

In a swine forelimb model, Ozer et al. (72) compared standard static cold preservation (4 degrees Celsius) for six hours to normothermic machine perfusion (27-32 degrees Celsius – room temperature) with autologous blood for 12 hours (**Table 5**). In contrast to cold preservation, which slows the metabolic rate leading to a depletion of ATP stores and acid-base disturbances, normothermic perfusion maintains normal metabolic rates (72). Further, normothermic perfusion appears to limit expression of hypoxia-related genes in comparison to static cold storage (75). In this study, animals receiving machine perfused grafts demonstrated adequate levels of microvascular blood flow and maintained near normal muscle contractility after transplantation up to 24 hours. In contrast, control animals demonstrated blood flow only in large vessels (radial and brachial arteries), without evidence for microvascular blood flow, and a steady decline in muscle contractility compared to the contralateral limb. Physiologic parameters were measured while VCA grafts were on the pump and in the recipient after transplantation, and no significant changes were observed among K+, pH, pO2, and pCO2. Lactate levels did increase while the graft was on pump but returned to normal levels in the recipient after transplantation. The same group then extended their perfusion time to 24 hours, instead of 12, with similar results (73). These results demonstrate that normothermic *ex vivo* machine perfusion of VCA grafts up to 24 hours is possible without major metabolic derangements and leads to superior functional outcomes compared to static cold storage (73, 74).

**Table 5:** *Ex vivo* perfusion.

Large Animal Model	VCA model	IS	Experimental Groups	Results/Conclusions	Reference
Swine	Orthotopic forelimb VCA	None	Group 1 – limbs were perfused for 12 hours using warm (27°C-32°C) autologous blood.	Warm perfusion with autologous blood led to acceptable levels of microvascular blood flow and near normal muscle contractility after transplantation up to 24 hours as well as stable physiological parameters: K+, pH, pO2, and pCO2.	(72)
			Group 2 – cold preservation control group, preserving limbs for 6 hours at 4°C. Following perfusion, limbs were transplanted into healthy swine		
Swine	Orthotopic forelimb VCA	None	Group 1 – (control) Limbs were perfused with heparin solution and preserved at 4°C for 6 hours.	Ex vivo warm perfusion of swine forelimb grafts for 12-24 hours improved microvascular blood flow and functional outcomes compared to static cold storage.	(73)
			Group 2 - Limbs were perfused with autologous blood at 27°C to 32°C for 24 hours. Following perfusion, limbs were transplanted into healthy swine.		
Swine	Forelimb VCA	None	Group 1 – Amputated limbs were perfused for 12 hours using an oxygenated colloid solution at 38°C containing washed RBCs;	Outcomes of extended normothermic preservation (EVNLP) of limbs up to 44 hours are not significantly different from short EVNLP (12 hours).	(74)
			Group 2 – Limbs were perfused until the vascular resistance increased (up to 44 hours). Contralateral forelimbs in each group were preserved at 4°C as a cold storage control group. Limbs were not transplanted.		

IS, immunosuppression; RBC, red blood cells; VCA, vascularized composite allotransplant.

## Discussion

The future of VCA appears to be at a crossroads (76). Historically, the United States Department of Defense (DOD) has been a major financial contributor to the field of VCA. Recently however, there has been a major push for insurance companies to cover the procedure and the ongoing care required, similar to solid organ transplantation (76). Given VCA’s perceived status as a non-vital transplant, insurance companies may be hesitant to provide lifelong reimbursement for the IS medications necessary. Continued advances in technology and immune strategies to improve VCA outcomes and reduce the IS burden will be an important step in moving the field of VCA forward. To this effect, large animal models of VCA will play a critical role in this endeavor. Large animal models of VCA have proven to share distinct technical, immunologic, and histopathological features with clinical solid organ and VCA transplantation. Because of these similarities, they will be useful in understanding the short- and long-term immunological barriers to VCA survival and optimizing novel immune modulating approaches to minimize IS, prevent VCA rejection, and expand the donor pool.

As in solid organ transplantation, swine and canine models appear to be most useful in contributing to mechanistic insights underlying tolerance induction and other proof of principle strategies (*ex vivo* perfusion). In contrast, preclinical NHP models more realistically reflect the clinical VCA experience in terms of acute and chronic alloimmune responses, IS effects, and histopathological features. Overcoming the immune barrier in preclinical NHP models of VCA will confer an increased chance of success when translated clinically. There is evidence that combining several of the strategies described here relative to each individual strategy alone may confer additional benefit in preclinical models of solid organ transplantation (77). However, these attempts should be approached cautiously in the setting of clinical VCA, given the nature of VCA as a life-enhancing graft rather than a life-saving option. Patient morbidity should be of utmost importance, and immune modulating strategies that result in over-immunosuppression of recipients should be avoided.

In conclusion, as clinical VCA expands to include more indications, the future of VCA research will undoubtedly expand in various directions. Key areas of research that will be important to moving the field forward include expanding the donor pool to potentially allow for HLA matching, overcoming the barrier of chronic rejection, and identifying strategies to minimize the need for chronic, high-dose IS. As described in this review, large animal models are well positioned to address these questions and continued funding of these models is imperative to support these studies.

## Author Contributions

AM: study design, literature search, wrote the paper, and edited the paper. RC: literature search, wrote the paper, and edited the paper. GM: wrote and edited the paper. CC: wrote and edited the paper. RT: study design, literature search, wrote the paper, and edited the paper. All authors contributed to the article and approved the submitted version.

## Conflict of Interest

The authors declare that the research was conducted in the absence of any commercial or financial relationships that could be construed as a potential conflict of interest.

## Abbreviations

VCA, vascularized composite tissue allotransplantation; HCT, Hematopoietic cell transplantation; BM, Bone Marrow; MHC, Major Histocompatibility Complex; GVHD, Graft *vs.* Host Disease; IS, Immunosuppression; NHP, Non-Human Primates; VBM, Vascularized Bone Marrow; MMF, mycophenolate mofetil; POD, Post operative day; HSC, Hemopoietic Stem Cells; ATG, Anti-thymocyte globulin; DLA, Dog Leukocyte Antigen; CMPSC, Cytokine mobilized peripheral stem cells; DSA, Donor Specific Antibody; CoB, Co-stimulation blockade; BM-MSCs, Bone marrow derived mesenchymal stem cells; DCregs, Dendritic regulatory cells; CyA, Cyclosporine; STSG, Split thickness skin graft; IRI, Ischemia reperfusion injury; H2S, Hydrogen Sulfide; UW, University of Wisconsin.
